# Productivity losses associated with tuberculosis deaths in the World Health Organization African region

**DOI:** 10.1186/s40249-016-0138-5

**Published:** 2016-06-01

**Authors:** Joses Muthuri Kirigia, Rosenabi Deborah Karimi Muthuri

**Affiliations:** African Sustainable Development Research Consortium (ASDRC), P.O. Box 6994 00100 GPO, Kenyatta Avenue, Nairobi, Kenya; Department of Psychology, School of Social Sciences and Humanities, United States International University, Nairobi, Kenya

## Abstract

**Background:**

In 2014, almost half of the global tuberculosis deaths occurred in the World Health Organization (WHO) African Region. Approximately 21.5 % of the 6 060 742 TB cases (new and relapse) reported to the WHO in 2014 were in the African Region. The specific objective of this study was to estimate future gross domestic product (GDP) losses associated with TB deaths in the African Region for use in advocating for better strategies to prevent and control tuberculosis.

**Methods:**

The cost-of-illness method was used to estimate non-health GDP losses associated with TB deaths. Future non-health GDP losses were discounted at 3 %. The analysis was conducted for three income groups of countries. One-way sensitivity analysis at 5 and 10 % discount rates was undertaken to assess the impact on the expected non-health GDP loss.

**Results:**

The 0.753 million tuberculosis deaths that occurred in the African Region in 2014 would be expected to decrease the future non-health GDP by International Dollars (Int$) 50.4 billion. Nearly 40.8, 46.7 and 12.5 % of that loss would come from high and upper-middle- countries or lower-middle- and low-income countries, respectively. The average total non-health GDP loss would be Int$66 872 per tuberculosis death. The average non-health GDP loss per TB death was Int$167 592 for Group 1, Int$69 808 for Group 2 and Int$21 513 for Group 3.

**Conclusion:**

Tuberculosis exerts a sizeable economic burden on the economies of the WHO AFR countries. This implies the need to strongly advocate for better strategies to prevent and control tuberculosis and to help countries end the epidemic of tuberculosis by 2030, as envisioned in the United Nations General Assembly resolution on Sustainable Development Goals (SDGs).

**Electronic supplementary material:**

The online version of this article (doi:10.1186/s40249-016-0138-5) contains supplementary material, which is available to authorized users.

## Multilingual abstracts

Please see Additional file 1 for translations of the abstract into the six official working languages of the United Nations.

## Background

The World Health Organization (WHO) estimates that the total number of deaths from tuberculosis (TB) worldwide was 1.514 million in 2014 [1]. Almost half of those deaths were from the WHO African Region. Approximately 21.5 % of the 6 060 742 TB cases (new and relapse) reported to the WHO in 2014 were in the African Region [1].

According to the WHO, TB is intimately linked to poverty, and the control of TB is ultimately a question of justice and human rights [2]. Failure to control TB (and other poverty-related diseases) is a consequence of the significant inequities in the distribution of wealth and health care both within and between countries [3–6]. In the African Region, the situation is exacerbated by the relatively high incidence and prevalence of co-infection of HIV/AIDS and TB and the growing problem of mycobacterial drug resistance [7–10].

The majority of TB deaths could have been prevented if the available preventive and treatment interventions were universally accessible to all those in need. Unfortunately, the coverage of those interventions is suboptimal in the African Region. For example, the BCG (Bacillus Calmette–Guérin) immunisation coverage among infants (aged 1 year) is between 50 and 70 % in 4 countries, 71–90 % in 17 countries, and 91 % and above in 26 countries [11]. The case detection rate for all forms of TB was 52 %, and the treatment success rate for new tuberculosis cases was 81 % [12]. In the absence of an effective vaccine for older ages, efforts to control the spread of TB will continue to rely on early diagnosis, directly observed therapy (DOTs) and public health infection control measures.

The prevention and control of TB is hampered by poor living conditions for vulnerable population groups and weak national health systems [12]. The national health systems lack capacities to assure universal access to TB prevention and control services for all those in need [13–16].

The situation calls for strong evidence-based advocacy for increased domestic and external investments into the fight against TB. One such evidence is the economic burden of TB. A retrospective cost-of-illness study in the United States estimated the 1991 direct expenditures for TB-related diagnosis and treatment to range from $515.7 million to $934.5 million [17]. Miller et al. estimated that in 2002, the 108 confirmed TB cases in Tarrant County (Texas, USA) cost a total of US$40 574 953 [18]. Rajbhandary et al. estimated the mean direct cost of treating a multi-drug-resistant (MDR-TB) patient in the United States to be US$45,000 [19].

Atun et al. estimated the mean cost of managing TB in Russia over 12 months to be US$572 per case [20]. Fløe et al. estimated the direct cost per TB patient to be €10 509 in Denmark [21]. Kik et al. estimated the direct and indirect costs of tuberculosis among immigrant patients in the Netherlands to be Euro 2 956 per TB patient [22]. The Diel et al. study in Germany estimated the total cost per MDR-TB/extensively drug-resistant (XDR-TB) case to be Euro 82 150 and Euro 108 733 per case, respectively [23]. In another study, Diel and colleagues performed a systematic review and revealed that the average cost of treatment of MDR-TB among 15 old European Union (EU countries plus Cyprus, Malta and Slovenia to be Euro 57 213 and Euro 24 166 [24]. White and Moore-Gillon estimated the mean direct cost of managing an MDR-TB patient in the United Kingdom to be £60 000 [25].

The Rajeswari et al. study in India estimated the total socio-economic impact of tuberculosis on patients and their families to be US$171 per case [26]. The Peabody et al. study in the Philippines estimated the combined economic losses due to premature tuberculosis-related mortality and morbidity to be US$145 million [27]. The Atif et al. study in Malaysia estimated the cost of tuberculosis treatment to be US$ 727.25 per patient [28].

The Mauch et al. study in Kenya revealed that a TB patient incurred a median direct cost of US$55.8 and an indirect cost of US$294.2 [29]. The Umar et al. study in Nigeria estimated the indirect cost due to pulmonary TB in patients receiving treatment to be US$517.98 and US$79.13 per hospitalised and non-hospitalized patient, respectively [30]. The Foster et al. study in South Africa estimated the mean total pre-treatment and treatment direct plus indirect costs incurred by respondents in accessing health care during TB diagnosis and treatment to be US$ 324.07 [31].

Except for Peabody et al. [27], none of the other studies included the economic losses due to premature tuberculosis-related mortality. In addition, to the best of our knowledge, no study has attempted to estimate the combined economic losses due to premature tuberculosis-related mortality for all 47 countries of the WHO African Region. Therefore, there is a dearth of evidence in the African Region on the economic burden of TB for use in advocacy for increased domestic and external investments in strengthening the national and local health systems to combat the spread of TB.

This paper attempts to answer the following question: What is the impact of TB deaths on the future non-health gross domestic product (GDP) in the WHO African Region? The specific objective of this study was to estimate the future GDP losses associated with TB deaths in the African Region to advocate for better strategies to prevent and control tuberculosis.

## Methods

### Cost-of-illness framework

Tuberculosis deaths result in future losses in the macroeconomic outputs of countries concerned with attrition of future labour and productivity, as well as an erosion of investments in human and physical capital formation [32]. In this paper, we employ a cost-of-illness model to estimate the non-health GDP losses attributable to TB-related deaths in the African Region.

Nattrass et al. [33] defines GDP as the value of the aggregate spending on all final goods and services. GDP is the sum of the private household consumption spending on final consumer goods (e.g., food, cloth, books, detergents) and services (e.g., health, education, tourism); central, regional and local government consumption spending on salaries and wages of civil servants and goods; private and public sectors producers investment spending on additional physical stock of capital (e.g., machinery, construction, vehicles) plus changes in the total value of inventories (unsold stocks); and net exports (i.e., exports minus imports).

Private consumption is the use of goods and services to directly satisfy an individual’s personal needs and wants [33]. Private consumption spending is funded from incomes earned by employees and self-employed people (e.g., farmers, entrepreneurs) and thus premature death of workers or self-employed people from tuberculosis (or any other cause) depletes household income and consumption. Death of those aged 0–14 years diminishes the quantity of future labour force and hence future household income and consumption.

Government consumption spending is financed largely through revenues from various forms of taxes, such as personal income tax, value-added tax, social security taxes, corporate taxes, and taxes on international trade and transactions [34]. Premature mortality due to tuberculosis (or any other cause) reduces the number of current and future tax payers and hence tax revenues available for government consumption spending and investment.

Investment spending is financed by savings, i.e., loanable funds [33, 34]. Once again, premature death from TB erodes a household’s current and future income and savings needed by investors. At times, the bereaved are forced by circumstances to sell assets and spend savings to pay for funeral expenses.

Agriculture is the main source of income and employment for the 62 % of the African Region population that lives in rural areas. In 2013, agriculture (including crops, forestry, hunting, and fishing, and livestock production) contributed 14.7 % to the GDP of Sub-Saharan Africa. However, the contribution varies from 2.3 % in South Africa to 58.2 % in the Central African Republic. Of 41 countries reporting, agriculture contributed to less than 10 % in 12 countries; 10–30 % in 16 countries; and 31–60 % in 13 countries [35]. Premature TB deaths would be expected to impact negatively on the agricultural and other sectors productivity.

According to WHO [32], the key ways through which tuberculosis deaths impact macroeconomic output include increased health expenditure, losses in labour and productivity and reduced investment in human and physical capital formation. This study uses a macroeconomic—or societal—perspective. The study’s scope is limited to market economy losses (GDP), its quantity of interest is the impact of tuberculosis deaths on non-health components of GDP, and its estimation method is the cost-of-illness model capturing the effects across all sectors of the economy [36].

The non-health GDP loss (*NHGDPLoss*) associated with tuberculosis deaths in a country is the sum of the potential non-health GDP loss due to tuberculosis deaths among those aged 0–14 (*NHGDPLoss*_0 − 14_), those aged 15–59 (*NHGDPLoss*_15 − 59_) and those aged 60 years and above (*NHGDPLoss*_60 ±_). Economic losses among the three age brackets were estimated to facilitate comparisons and to avail information for use in advocacy for an increase in investments against tuberculosis, and the growing challenge of antimicrobial resistance in the region.

The non-health GDP loss associated with tuberculosis deaths among persons of a specific age group is the product of the total discounted years of life lost, per capita non-health GDP in purchasing power parity (PPP) and the total number of tuberculosis deaths. Each country’s discounted total non-health GDP loss attributable to tuberculosis deaths was estimated using eqs. (1), (2), (3) and (4) presented below [37].

1 $$ NHGDPLoss=\left( NHGDPLos{s}_{0-14}+ NHGDPLos{s}_{15-59}+ NHGDPLos{s}_{60\pm}\right) $$

2 $$ \begin{array}{l} NHGDPLos{s}_{0-14}={\displaystyle \sum_{t=1}^n\left\{\left[1/{\left(1+r\right)}^t\right]\right.}\times \left[ NHGDPP{C}_{Int\$}\right]\times \left.\left[TB{D}_{0-15}\right]\right\}=\\ {}\kern2.16em \left\{\left[1/{\left(1+r\right)}^1\right]\right.\times \left[ NHGDPP{C}_{Int\$}\right]\times \left.\left[TB{D}_{0-14}\right]\right\}+\\ {}\kern2.16em \left\{\left[1/{\left(1+r\right)}^2\right]\right.\times \left[ NHGDPP{C}_{Int\$}\right]\times \left.\left[TB{D}_{0-14}\right]\right\}+\dots +\\ {}\kern2.16em \left\{\left[1/{\left(1+r\right)}^n\right]\right.\times \left[ NHGDPP{C}_{Int\$}\right]\times \left.\left[TB{D}_{0-14}\right]\right\}\kern0.24em \end{array} $$

3 $$ \begin{array}{l} NHGDPLos{s}_{15-59}={\displaystyle \sum_{t=1}^n\left\{\left[1/{\left(1+r\right)}^t\right]\right.}\times \left[ NHGDPP{C}_{Int\$}\right]\times \left.\left[TB{D}_{15-59}\right]\right\}=\\ {}\kern2.16em \left\{\left[1/{\left(1+r\right)}^1\right]\right.\times \left[ NHGDPP{C}_{Int\$}\right]\times \left.\left[TB{D}_{15-59}\right]\right\}+\\ {}\kern2.16em \left\{\left[1/{\left(1+r\right)}^2\right]\right.\times \left[ NHGDPP{C}_{Int\$}\right]\times \left.\left[TB{D}_{15-59}\right]\right\}+\dots +\\ {}\kern2.16em \left\{\left[1/{\left(1+r\right)}^n\right]\right.\times \left[ NHGDPP{C}_{Int\$}\right]\times \left.\left[TB{D}_{15-59}\right]\right\}\end{array} $$

4 $$ \begin{array}{l} NHGDPLos{s}_{60\pm }={\displaystyle \sum_{t=1}^n\left\{\left[1/{\left(1+r\right)}^t\right]\right.}\times \left[ NHGDPP{C}_{Int\$}\right]\times \left.\left[TB{D}_{60\pm}\right]\right\}=\\ {}\kern2.16em \left\{\left[1/{\left(1+r\right)}^1\right]\right.\times \left[ NHGDPP{C}_{Int\$}\right]\times \left.\left[TB{D}_{60\pm}\right]\right\}+\\ {}\kern2.16em \left\{\left[1/{\left(1+r\right)}^2\right]\right.\times \left[ NHGDPP{C}_{Int\$}\right]\times \left.\left[TB{D}_{60\pm}\right]\right\}+\dots +\\ {}\kern2.16em \left\{\left[1/{\left(1+r\right)}^n\right]\right.\times \left[ NHGDPP{C}_{Int\$}\right]\times \left.\left[TB{D}_{60\pm}\right]\right\}\end{array} $$

where 1/(1 + *r*)^*t*^ is the discount factor; *r* is the rate of discount of future losses; $$ {\displaystyle \sum_{t=1}^n} $$ is the summation from year *t* to *n*; *t* is the first year of life lost, and *n* is the final year of the total number of years of life lost per tuberculosis death, which is obtained by subtracting the average age at death (AAD) for tuberculosis-related causes from each country’s average life expectancy at birth. *NHGDPPC*_*Int$*_ is the per capita non-health gross domestic product in purchasing power parity (PPP), which is obtained by subtracting the per capita total health expenditure (*PCTHE*) from the per capita GDP (*GDPPC*_*Int$*_). *TBD*_0 − 14_ is the total tuberculosis deaths between the ages of 0–14 years in country *k* in 2013; *TBD*_15 − 59_ is the total tuberculosis deaths between the age of 15–59 years in country *k* in 2013; and *TBD*_60 ±_ is the total tuberculosis deaths for ages 60 years and above in country *k* in 2013. We used 2013 as the base year to which losses occurring in future years were discounted. As explained by Kirigia [38], Drummond et al. [39] and Curry and Weiss [40], the discount factor applied to the GDP losses of different years then depends on both the discount rate (r) and the number of years (t) over which the discounting is conducted.

The non-health GDP per capita in purchasing power parity for each of the 47 countries in the WHO African Region was calculated by subtracting the per capita total health expenditure from the per capita GDP.

#### Illustration of calculation of loss in total non-health GDP

The example below presents a calculation of the tuberculosis death-related loss in non-health GDP using the actual information on Nigeria:

- Total tuberculosis deaths in Nigeria in 2014 = 250 000
- Proportion of deaths among those aged 0–14 years = 0.125255548607164
- Proportion of deaths among those aged 15–59 years = 0.774170807391794
- Proportion of deaths among those aged 60+ years = 0.100573644001042
- *TBD*_0 − 14_ = 250000 × 0.125255548607164 = 31313.887151791
- *TMD*_15 − 59_ = 250000 × 0.774170807391794 = 193542.701847949
- *TMD*_60 +_ = 250000 × 0.100573644001042 = 25143.4110002605
- Average age at death among those aged 0–14 years (*AAD*_0 − 14_), i.e., (0 + 14)/2 = 7 years
- Number of years needed, in addition to *AAD*_0 − 14_, to reach the legal minimum age for employment of 15 years (*Age*_0 − 14*Min*_), i.e., 7 years.
- Average age at death among those aged 15–59 years (*AAD*_15 − 59_), i.e., (15 + 59)/2 = 37 years
- Average age at death among those aged 60 years and older (*AAD*_60 ±_), i.e., 60 years
- Nigeria’s average life expectancy at birth (LE) = 55 years
- Per capita gross domestic product (*GDPPC*_*Int$*_ ) = *Int$*5756.271
- Per capita total expenditure on health (*PCTHE*) = *Int$*207
- *NHGDPPC* = *GDPPC*_*Int$*_ − *PCTHE* = *Int$* 5756.271 − *Int$*207 = *Int$* 5550
- Discount rate (*r*) = 3 %
- Undiscounted years of life lost in the group aged 0–14 years (*YLL*_0 − 14_) = *LE* – (*AAD*_0 − 14_ + *Age*_0 − 14*Min*_ ) = 55 – (7 + 7) = 41 *years*
- Discounted years of life lost in the group aged 0–14 years (*DYLL*_0 − 14_) = 23.4123999749577
- Undiscounted years of life lost in the group aged 15–59 years (*YLL*_15 − 59_) = *LE* − *AAD*_15 − 59_ = 55 – 37 = 18 *years*
- Discounted years of life lost in the group aged 15–59 years (*DYLL*_15 − 59_) = 13.7535130794572
- Undiscounted years of life lost in the group aged 60+ years (*YLL*6_60 ±_) = *LE* − *AAD*_60 ±_ = 0 (because the Nigeria average LE of 55 years is less than 60 years, we assumed that years of life lost within this age group are zero. However, this assumption is adjusted in the sensitivity analysis where we re-estimate the model using highest life expectancy in the region, i.e., 75 years in Cape Verde).
- Discounted years of life lost in the group aged 60+ years (*DYLL*_60 +_) equals zero for reason explained in ‘t’ above.
- *NHGDPLoss*_0 − 14_ = *Discounted YLL*_0 − 15_ × *NHGDPPC*_*Int$*_ × *TBD*_0 − 14_ = 23.4123999749577 × 5550 × 31313.887151791 = *Int$*4 068 889 541.76473
- *NHGDPLoss*_15 − 59_ = *Discounted YLL*_15 − 59_ × *NHGDPPC*_*Int$*_ × *TBD*_15 − 59_ = 13.7535130794572 × 5550 × 193542.701847949 = *Int$*14 773 501 051.2108
- *NHGDPLoss*_60 ±_ = *Discounted YLL*_60 ±_ × *NHGDPPC*_*Int$*_ × *TBD*_60 ±_ = 0 × 5550 × 25143.4110002605 = *Int$*0.
- *NHGDPLoss* = (*NHGDPLoss*_0 − 14_ + *NHGDPLoss*_15 − 59_ + *NHGDPLoss*_60 ±_) = *Int$*4 068 889 541.76473 + *Int$*14 773 501 051.2108 + *Int$*0 = *Int$*18 842 390 592.9755.

### Sensitivity analysis

A discount rate of 3 % was used because it is commonly used in cost-of-illness studies [41, 42], burden of disease studies [43, 44] and WHO health systems’ performance assessment [45]. However, a one-way sensitivity analysis was conducted at 5 and 10 % discount rates to test the effect of the discount rate on the overall total expected non-health GDP loss estimate.

The study used 7 years (a simple average) as the average age at death for the 0–14 age bracket; 37 years for the 15–59 age bracket; and 60 years for the 60 years and above. Because the legal minimum working age limit is 15 years [46], we considered only the years above 14 years when calculating the productive years of life lost for the 0–14 age bracket. A sensitivity analysis was conducted to determine the effect of age on the overall total non-health GDP loss estimate. The model was re-estimated assuming an average age at death of 0 years for the 0–14 age bracket; an average age at death of 15 years for the 15–59 age bracket; and each country’s average life expectancy as the average age at death for the age bracket of 60 years and above, while simultaneously assuming the African Region’s maximum life expectancy of 75 years (i.e., life expectancy for Cape Verde).

### Data sources and analysis

The data used to estimate eqs. 1, 2, 3 and 4 were obtained from following sources: the life expectancy at birth data were taken from WHO World Health Statistics 2015 [12]; the proportions of deaths occurring in the three age groups were from the WHO mortality and burden of disease estimates for WHO member states in 2008 [43]; the total tuberculosis deaths were taken from the WHO World Tuberculosis Report 2015 [1]; the per capita gross domestic product in purchasing power parity (PPP) values were from the International Monetary Fund database [47]; and per capita total health expenditure data were from the World Health Statistics 2015 [12].

The formulas in eqs. (1), (2), (3) and (4) were used to estimate the non-health GDP losses and were built in an Excel spreadsheet. For the analysis, the countries were organised into three economic groups, as shown in Table 1, with high- and upper-middle-income countries in Group 1, lower-middle-income countries in Group 2 and low-income countries in Group 3. A calculation for the countries by income group was meant to facilitate comparisons.

**Table 1:** Economic classification of WHO African Region Countries in 2013

Group	GNI per capita (US$)	Countries
Group 1: High income and upper-middle income	> = 4 086	Algeria, Angola, Botswana, Equatorial Guinea, Gabon, Mauritius, Namibia, Seychelles, South Africa (9)
Group 2: Lower-middle income	1 036–4 085	Cameroon, Cape Verde, Congo, Cote d’Ivoire, Ghana, Kenya, Lesotho, Mauritania, Nigeria, São Tome and Principe, Senegal, Swaziland, Zambia (13)
Group 3: Low income	1 035 or less	Benin, Burkina Faso, Burundi, Central African Republic, Chad, Comoros, DRC, Eritrea, Ethiopia, The Gambia, Guinea, Guinea-Bissau, Liberia, Madagascar, Malawi, Mali, Mozambique, Niger, Rwanda, Sierra Leone, South Sudan, Tanzania, Togo, Uganda, Zimbabwe (25)

### Ethical clearance

The study did not require WHO/AFRO Ethics Review Committee approval because it did not involve human subjects. It relied entirely on data from published sources.

## Results

Table 2 presents the WHO African Region’s population and tuberculosis deaths by economic group in 2014. Of the total of 753 423 tuberculosis deaths that occurred, 16.26 % belonged to the high- and upper-middle-income countries (Group 1), 44.73 % to the lower-middle-income countries (Group 2) and 39.01 % to the low-income countries (Group 1).

**Table 2:** Population and tuberculosis deaths by economic group in WHO African Region countries

Group/economic class	(A) Population in 2013	(B) Tuberculosis Deaths in 2014	Percentage = (B/A)x100
Group 1: High income & upper-middle income	121 546 000	122 526	0.101
Group 2: Lower-middle income	331 470 000	337 009	0.102
Group 3: Low income	478 356 000	293 888	0.061
TOTAL	931 372 000	753 423	0.081

*Source*: WHO [1, 12]

### Non-health GDP loss attributable to tuberculosis deaths

The 0.753 million tuberculosis deaths that occurred in the African Region in 2014 would be expected to decrease future non-health GDP by Int$50,382,574,953 (Table 3). Nearly 40.8 % of the loss would be represented by Group 1 countries, 46.7 % by group 2 and 12.5 % by group 3. The interquartile range of the median GDP loss by country is Int$440,387,653. The potential loss of future discounted non-health GDP would vary widely, from Int$0 in Seychelles to Int$18.84 billion in Nigeria.

**Table 3:** Discounted values of future non-health GDP losses from tuberculosis deaths among WHO African Region countries in 2014 (2013, Int$ or PPP)

Countries	International Dollars (PPP)	Percentage
Algeria	1 245 265 365	2.47
Angola	1 283 658 670	2.55
Benin	36 750 328	0.07
Botswana	405 550 047	0.80
Burkina Faso	52 205 284	0.10
Burundi	42 896 468	0.09
Cameroon	671 947 468	1.33
Cape Verde	23 398 102	0.05
Central African Republic	36 287 667	0.07
Chad	160 379 659	0.32
Comoros	1 492 351	0.00
Congo	432 543 715	0.86
Cote D’Ivoire	229 438 028	0.46
DRC	426 514 828	0.85
Equatorial Guinea	64 069 641	0.13
Eritrea	18 720 135	0.04
Ethiopia	870 327 842	1.73
Gabon	392 992 668	0.78
Gambia	12 332 470	0.02
Ghana	934 667 549	1.86
Guinea	87 848 382	0.17
Guinea-Bissau	44 826 660	0.09
Kenya	870 294 829	1.73
Lesotho	149 807 038	0.30
Liberia	43 220 969	0.09
Madagascar	301 036 739	0.60
Malawi	140 425 230	0.28
Mali	44 430 032	0.09
Mauritania	61 852 822	0.12
Mauritius	8 191 726	0.02
Mozambique	719 984 725	1.43
Namibia	501 033 284	0.99
Niger	54 836 009	0.11
Nigeria	18 841 273 630	37.40
Rwanda	25 108 682	0.05
Sao Tome & Principe	1 007 272	0.00
Senegal	131 075 954	0.26
Seychelles	–	0.00
Sierra Leone	46 928 838	0.09
South Africa	16 633 567 089	33.01
South Sudan	119 338 372	0.24
Swaziland	233 669 870	0.46
Tanzania	2 538 873 431	5.04
Togo	17 528 131	0.03
Uganda	289 994 353	0.58
Zambia	944 859 661	1.88
Zimbabwe	190 122 940	0.38
Total loss (Int$)	50 382 574 953	100.00

### Non-health GDP loss in Group 1 countries

**Fig. 1:**
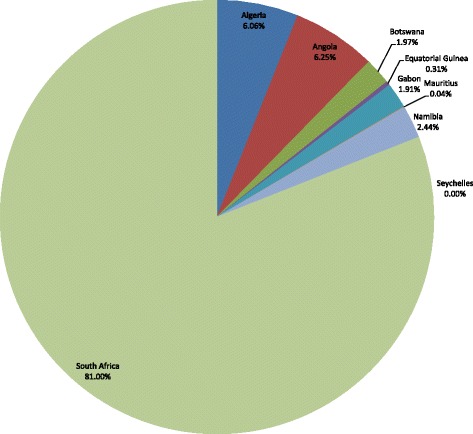
Non-health GDP loss in Group 1 due to tuberculosis deaths in high-income and upper-middle- income countries of the WHO African Region, 2013

The 122 526 TB deaths in Group 1 countries are expected to result in a total loss of Int$20 534 328 490 in non-health GDP in 2013, which is equivalent to 1.36 % of the group’s total GDP. The total productivity loss varied importantly, from Int$0 in Seychelles to Int$16.6 billion in South Africa. Figure 1 displays the distribution of the total non-health GDP across the nine high and upper-middle income countries in Group 1. Approximately 81 % of the expected loss in Group 1 was represented by South Africa.

### Non-health GDP loss in Group 2 countries

**Fig. 2:**
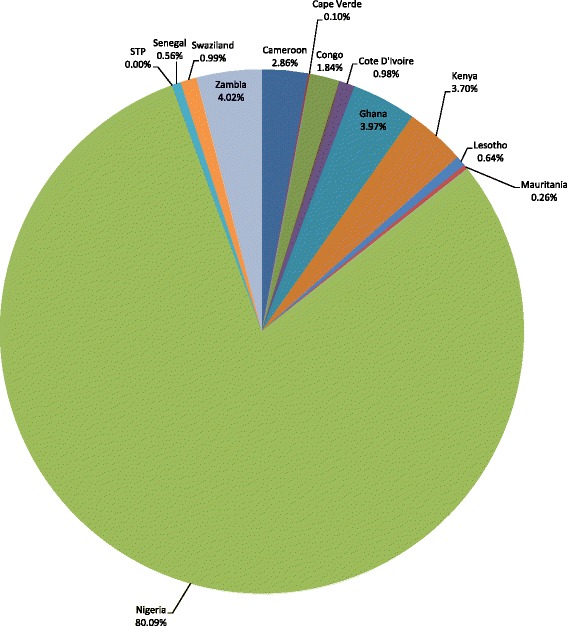
Non-health GDP loss in Group 2 due to due to tuberculosis deaths in lower-middle-income countries of the WHO African Region, 2013

The 337 009 TB deaths in Group 2 countries resulted in an expected total loss of Int$23 525 835 936 in non-health GDP, or 1.6 % of the group’s total GDP. The loss was wide-ranging, from Int$1 007 272 in São Tomé and Príncipe to Int$18 841 273 630 in Nigeria. Figure 2 presents the distribution of the total non-health GDP loss across the 13 lower-middle income countries in Group 2. Approximately 80.1 % of the loss in Group 2 was represented by Nigeria.

### Non-health GDP loss in Group 3 countries

**Fig. 3:**
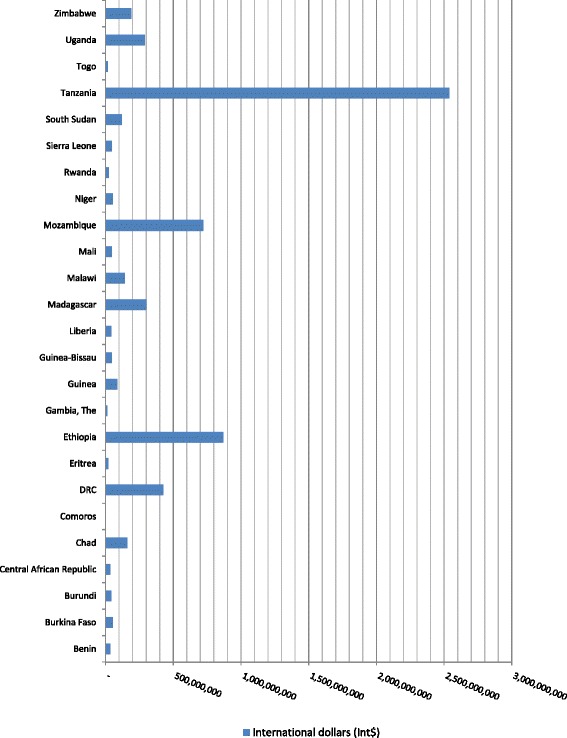
Non-health GDP loss in Group 3 due to tuberculosis deaths in low-income countries of the WHO African Region, 2013

The 293 888 TB deaths that occurred among Group 3 countries in 2013 resulted in a total expected loss in non-health GDP of Int$6 322 410 528, which is equivalent to 0.91 % of the group’s total GDP. The expected loss varied from Int$1.5 million in Comoros to Int$2.54 billion in Tanzania. Figure 3 shows the distribution of the total non-health GDP loss across the 25 low-income countries in Group 3. The Democratic Republic of the Congo (DRC), Ethiopia, Madagascar, Mozambique and Tanzania collectively incurred 76.8 % of the expected loss in this group. In spite of the fact that Group 3 TB deaths were 2.4 times those of Group 1, the non-health GDP loss of Group 1 was 3.2 times higher than that of Group 3 because Group 1 had a higher per capita GDP.

### Average non-health GDP losses

Table 4 shows the average non-health GDP losses per TB death and per person in population for the 47 countries. The average non-health GDP lost per TB death was Int$167,592 for Group 1, Int$69 808 for Group 2 and Int$21,513 for Group 3. The average non-health GDP loss per person in the population was Int$168.9 for Group 1, Int$71 for group 2 and Int$13.2 for Group 3. The average non-health GDP lost per TB death was slightly more than two times that for Group 2 and about eight times that for Group 3.

**Table 4:** Discounted values of future non-health GDP lost due to TB deaths in 2014 by economic group (in 2013 international dollars)

Cost item	Group 1 (Int$)	Group 2 (Int$)	Group 3 (Int$)	Grand total Cost (Int$)
Total cost of TB deaths	20 534 328 490	23 525 835 936	6 322 410 528	50 382 574 953
Average cost per TB death	167 592	69 808	21 513	66 872
Average cost per person in population	168.9	71.0	13.2	54.1
% of Grand Total	40.8	46.7	12.5	100

### Sensitivity analysis

Employing a 5 % discount rate reduced the total expected non-health GDP loss by Int$9.703 billion (19.26 %) and the average non-health cost per TB death by Int$12 878. Whereas, application of a 10 % discount rate reduced the overall total non-health GDP loss by Int$23 851 884 140 (47.34 %) and the average non-health GDP loss per TB death by Int$31 658.

The use of the average age at death of 0 years for the 0–14 age bracket; 15 years for the 15–59 age bracket; and each country’s average life expectancy as the average age at death for the age bracket of 60 years and above, while simultaneously assuming the region’s maximum life expectancy of 75 years, raised the total non-health GDP loss by Int$28.4 billion, which is a 56.3 % increase. This also increased the average non-health GDP loss per TB deaths by Int$37,649. Because the non-health GDP loss also seems to partially depend on the average age used for the onset of TB deaths, there is a need for epidemiological research into the age distribution of TB deaths.

## Discussion

The estimated total expected non-health GDP loss ascribed to TB deaths of Int$50.4 billion is approximately 1.37 % of the collective GDP of the 47 WHO African Region member states. This estimate signifies the expected loss in potential GDP in the future from the 753,423 TB deaths, which is revalued relative to the base year of 2013. The sensitivity analysis revealed that the size of the total non-health GDP loss partially depends on the discount rate used and the average age used for the onset of TB deaths. The latter implies that there is a need for epidemiological research into the age distribution of TB deaths.

The Group 3 (low income) countries are the home of 51.4 % of the African Region population, incurred 39 % of TB deaths, and bore only 12.5 % of non-health GDP losses associated with TB deaths in the region. On the other hand, even though Group 1 (high income and upper-middle income) countries have only 13.1 % of the regional population and incurred only 16.3 % of TB deaths (probably due to better living and working conditions), it bore 40.8 % of the non-health GDP losses associated with TB in the region. This is attributed to the fact that the Group 1 per capita income of Int$9,257 is eight times higher than that of Group 3 countries of Int$1,131. This implies that even though the TB disease burden is lower in Group 1 vis-a-vis Group 3, it should not be a reason for complacency because the negative impact on Group 1 economies is quite sizeable.

As mentioned in the Background, there is a worldwide paucity of studies that estimate the economic losses due to premature mortality from TB. Peabody et al. estimated the combined annual income loss due to TB morbidity and premature mortality to be US$145 million in the Philippines in 1997, of which US$32 million (22.1 %) was attributed to premature mortality [27]. Hickson estimated the magnitude of the decline in the mortality and morbidity burden of TB at 104,425 life years, valued at US$127 billion in England and Wales. Out of the latter loss, $71 billion (55.9 %) was attributed to TB mortality [48]. The median GDP loss per country in the African Region was Int$140.4 million in 2013, which confirms that premature mortality from TB lowers a country’s GDP.

Cognizant of the correlation between health and economic development, the UN General Assembly in 2015 adopted a development agenda whose sustainable development goal (SDG) 3 focuses on ensuring healthy lives and promoting well-being for all people at all ages [49]. Target 3.3 focuses on ending the epidemics of AIDS, tuberculosis, malaria and neglected tropical diseases and combating hepatitis, water-borne diseases and other communicable diseases by 2030. The Sixty-Seventh World Health Assembly resolution, WHA67.1, adopted the global strategy and targets for TB prevention, care and control after 2015 [50]. The strategy provides detailed guidance to member states on key interventions for eliminating TB by 2035; some of which include the following: early diagnosis and treatment using DOTS; treatment of all people with multi-drug-resistant TB; and antiretroviral therapy for HIV-positive TB patients with tuberculosis/HIV activities [51].

One may ask whether those interventions are economically viable. Korenromp et al. [52] projected that in the African Region, the cost of diagnosing and treating one TB patient under DOTS would be US$503, with an additional cost incurred if the patient has multi-drug resistance (MDR) TB that would be US $4 315. Other additional costs would be incurred if the patent is HIV-positive and receives antiretroviral therapy (ART) for the duration of a 6-month DOTS course, which would be US$236 in 2010. As shown in Table 5, if we inflate those 2010 costs by 3 % per year over a period of 3 years (to 2013) and sum them, then we obtain a total cost of US$1 975 738 381, which when discounted at 3 % comes to $1 918 192 602. Dividing the GDP loss (which is potential savings) of $50.4 billion by the cost of TB interventions ($1.92 billion) yields a benefit-cost ratio (BCR) of 26.2. This means that policymakers can expect $26.2 in benefits for every $1 invested in the three TB interventions. Therefore, since the BCR is greater than 1, this means the benefits outweigh the costs and the investment into the three interventions for TB patients should be considered worthwhile.

**Table 5:** Total cost of TB interventions and the benefit-cost ratio for the African Region

Variables	Number of TB cases^a^	Cost per TB patient ($)^b^	Sub-Total Cost ($)
TB Incidence (including HIV)	2 700 000	549.6417	1 484 032 538.70
TB Incidence (HIV-positive)	870 000	257.8836	224 358 707.64
MDR-TB	56 700.00	4715.117	267 347 134.18
Undiscounted total cost (USD)			1 975 738 381
(A). Discounted total cost (Int$) at a 3 % rate			1 918 192 602.45
(B). GDP Loss (potential saving)			50 382 574 953
Benefit/cost ratio, i.e., (B)/(A)			26.2656497

*Source*: ^a^WHO [1]; ^b^Korenromp et al. [52]

The sizeable economic losses attributable to premature TB-related mortality imply an urgent need for governments (in collaboration with the Regional Economic Communities, the private sector, the civil society, Global Health Initiatives and development partners) to fully implement the global End TB Strategy to eliminate premature mortality from TB. The full implementation of the strategy to curb the TB disease burden and attenuate the related economic losses has high-level political support contained in the decisions and resolutions on TB from the Organization of African Unity/African Union [53–56], the WHO Regional Committee for Africa [57–60], the World Health Assembly [50, 61–64] and the United Nations General Assembly [65, 66].

### Limitations of the study

This study has a number of limitations. First, it focuses only on the effects that TB-related premature mortality has on the economy. It does not include the cost of absence from work and reduced labour performance/productivity due to prolonged periods of sickness. We omitted direct costs, including health care cost of treating ordinary TB cases and those resulting from longer hospital stays for individuals with resistant infections, e.g., MDR-TB and XDR-TB.

Second, the GDP per capita gives no indication about how available resources are distributed across people and households. For instance, the average income per capita might remain unchanged while the distribution of income changes, which has implications for the typical household [67].

Third, the GDP only captures economic activities associated with market transactions. Its calculation omits the value of full-time homemakers (domestic labour). For example, the value of labour of women who choose to stay at home doing house work and raising children is omitted [68].

Fourth, GDP does not include the cost of production or consumption processes externalities such as pollution, environmental degradation and costs of substance abuse (e.g., alcohol, smoking) [68].

Finally, loss of human life due to tuberculosis has an effect on the well-being of the bereaved family members that goes well beyond the loss of incomes to which it gives rise [69]. Some of that effect includes psychological pain of losing a loved one; the stress and anxiety of losing a caretaker or a breadwinner; and negative impact on the children’s nutrition status and education when a parent dies.

## Conclusion

This paper sought to contribute to the literature on the economic burden of TB. The 47 WHO African Region Member States lost 1.37 % of their combined GDP due to the 753 423 TB deaths in 2014. That is a sizeable loss in a Region where 47 % of the population lives on less than one international dollar per day [12]. Approximately 75.86 % of the loss was represented by those aged 15–59 years, which is the most productive age bracket.

The fact that a premature mortality resulting from TB lowers the GDP implies that the governments of African countries in collaboration with the Regional Economic Communities, private sector, the civil society, Global Health Initiatives and development partners ought to support full implementation of the Global End TB Strategy.

The economic evidence contained in this paper is only one argument for the universal coverage of public health interventions to end morbidity and premature mortality from TB. The literature is replete with other arguments such as the contagious nature of TB and its threat to global health security [70], the growing burden of MDR-TB and XDR-TB [1, 71], comorbidity of HIV and TB [72], sub-optimal performance of national TB programmes [73] and human rights (social justice) considerations [74].

## Additional file

Additional file 1: Multilingual abstract in the six official working languages of the United Nations (PDF 330 kb)

## Abbreviations

AAD: average age at death
AAD= > 60: average age at death for those aged 60 years and above
AAD0–14: average age at death for those aged 0–14 years
AAD15–59: average age at death for those aged 15–59 years
DOTS: directly observed treatment, short-course
DYLL: discounted years of life lost
DYLL_±60_: discounted years of life lost in the group aged 60 years
DYLL_0-14_: discounted years of life lost in the group aged 0–14 years
DYLL_15-59_: discounted years of life lost in the group aged 15–59 years
GDP: gross domestic product
GDPPC_Int$_: Per capita GDP in international dollars
Int$: international dollars
LE: life expectancy at birth
MDR-TB: multi-drug-resistant TB
N: final year of the total number of years of life lost per death associated with TB
NHGDPC_Int$_: per capita non-health GDP in PPP
NHGDPLoss: non-health GDP loss
NHGDPLoss_=>60_: non-health GDP loss in the 60 years and above age group
NHGDPLoss_0–14_: non-health GDP loss in the 0–14 age group
NHGDPLoss_15–59_: non-health GDP loss in the 15–59 age group
PCTHE: per capita total health expenditure
PPP: purchasing power parity
PYLL: productive years of life lost
R: rate of discount of future losses
SDG: sustainable development goals
t: first year of life lost
TB: tuberculosis
TBD: total number of deaths associated with TB
TBD_0-14_: total tuberculosis deaths associated with TB in the 0–14 age group
TBD_15-59_: total tuberculosis deaths associated with TB in the 15–59 age group
TBD_60±_: total tuberculosis deaths associated with TB in the 60 years and above age group
WHO: World Health Organization
XDR-TB: extensively drug-resistant TB
YLL_0-14_: undiscounted years of life lost in the group aged 0–14 years
YLL_15-59_: undiscounted years of life lost in the group aged 15–59 years
YLL_±60_: undiscounted years of life lost in the group aged 60 years and above
