# Supplementary material for: Productivity losses associated with tuberculosis deaths in the World Health Organization African region
Source: Infect Dis Poverty. 2016 Jun 1;5:43. doi: 10.1186/s40249-016-0138-5 (PMC4888542; doi:10.1186/s40249-016-0138-5)
Supplement: Additional file 1: — Multilingual abstract in the six official working languages of the United Nations (PDF 330 kb) [file 40249_2016_138_MOESM1_ESM.pdf]

Translation of the abstract into the six official working languages of the United Nations

خسائر الإنتاجية المرتبطة بوفيات مرضى السل في الإقليم الإفريقي التابع لمنظمة الصحة العالمية

جوسيس موثوري كيريجيا، روسينابي ديبورا كاريمي موثوري

ملخص

خلفية: حدثت نصف وفيات مرض السل تقريبًا في عام 2014 على مستوى العالم في الإقليم الإفريقي التابع لمنظمة الصحة العالمية (WHO). فحوالي 21.5٪ من الحالات (الجديدة والمنتكسة) التي أُبلِغ عنها لمنظمة الصحة العالمية في عام 2014، والبالغ عددها 742 060 6، كانت في الإقليم الإفريقي. وكان هدف هذه الدراسة بالتحديد هو تقدير خسائر الناتج المحلي الإجمالي (GDP) المستقبلية المرتبطة بوفيات مرضى السل في الإقليم الإفريقي، وذلك لاستخدامها في دعم استراتيجيات أفضل من أجل الوقاية من مرض السل والسيطرة عليه.

الوسائل: استُخدمت وسيلة تكلفة المرض لتقدير خسائر الناتج المحلي الإجمالي التي ليس لها صلة بقطاع الصحة والمرتبطة بوفيات مرضى السل. خُفِّضت خسائر الناتج المحلي الإجمالي المستقبلية التي ليس لها صلة بقطاع الصحة بنسبة 3%. وأُجري التحليل لثلاث مجموعات دخل من البلدان. وأُجري تحليل حساسية أحادي المتغير عند معدلي الخفض بنسبة 5% و10% لتقييم التأثير على خسائر الناتج المحلي الإجمالي المتوقعة التي ليس لها صلة بقطاع الصحة.

النتائج: يُتوقع أن يتسبب عدد وفيات مرضى السل التي حدثت في الإقليم الإفريقي في عام 2014، وقُدرت بـ0.753 مليون حالة وفاة، في خفض الناتج المحلي الإجمالي المستقبلي الذي ليس له صلة بقطاع الصحة بمقدار 50.4 مليار دولار دولي. وستنتج حوالي 40.8% و46.7% و12.5% من هذه الخسارة من البلدان ذات الدخل العالي والدخل المتوسط العالي أو البلدان ذات الدخل المتوسط المنخفض والدخل المنخفض على التوالي. وسيكون متوسط إجمالي خسائر الناتج المحلي الإجمالي التي ليس لها صلة بقطاع الصحة 872 66 دولارًا دوليًا لكل حالة وفاة بمرض السل. وكان متوسط خسائر الناتج المحلي الإجمالي لكل حالة وفاة بمرض السل 592 167 دولارًا دوليًا للمجموعة الأولى، و808 69 دولار دولي للمجموعة الثانية، و513 21 دولار دولي للمجموعة الثالثة.

الاستنتاج: يمثل مرض السل عبئًا اقتصاديًا ضخمًا على اقتصاد بلدان الإقليم الإفريقي التابع لمنظمة الصحة العالمية. مما يوحي بأهمية الدعم القوي لاستراتيجيات أفضل للوقاية من مرض السل والسيطرة عليه، ولمساعدة البلدان على القضاء على وباء السل بحلول عام 2030، بحسب تصور قرار الجمعية العامة للأمم المتحدة بشأن أهداف التنمية المستدامة (SDGs).

Translated from English version into Arabic by Doha Ahmad, through

世界卫生组织非洲地区结核病死亡导致的生产力损失

Joses Muthuri Kirigia，Rosenabi Deborah Karimi Muthuri

摘要：

引言：2014 年约有 21.5%的结核病病例（包括新发和复发）来自非洲地区，而几乎全球一半的结核病死亡病例来自该地区。本研究的主要目的是估算非洲地区将来由结核病死亡导致的国内生产总值(GDP)损失，从而提倡使用更好的策略来预防和控制结核病。

方法：采用疾病-成本方法来估算由结核病导致的非健康 GDP 损失，未来非健康 GDP 损失折扣为 3%。通过分析 3 个收入群体国家，采用单向灵敏度分析，以折现率为 5%和 10%来评估预期非健康 GDP 损失的影响。

结果：2014 年非洲地区的 75.3 万例结核病死亡病例能降低未来 50 亿国际元的非健康 GDP。接近 40.8%、46.7%和 12.5%的损失分别来自高收入和中高收入国家（第一分组）、中低收入国家（第二分组）和低收入国家（第三分组）。每个结核病死亡造成的平均非健康 GDP 损失为 66 872 国际元。在第一、第二和第三分组国家中，结核病死亡造成的平均非健康 GDP 损失分别为 167 592、69 808 和 21 513 国际元。

结论：结核病给 WHO 非洲地区国家造成相当大的经济负担。这表明需要提倡使用更好的策略来预防和控制结核病，并帮助这些国家在 2030 年前阻断其流行，正如在联合国大会上关于可持续发展目标决议中所展望的一样。

Translated from English version into Chinese by Feng Xin-Yu, edited by Yang Pin, through

Pertes de productivité associées aux décès imputables à la tuberculose dans la région Afrique de l’Organisation Mondiale de la Santé

Joses Muthuri Kirigia，Rosenabi Deborah Karimi Muthuri

Résumé

Contexte: En 2014, près de la moitié des décès imputables à la tuberculose dans le monde se sont produits dans la région Afrique de l'Organisation Mondiale de la Santé. Près de 21,5 % des 6 060 742 cas de TB (nouveaux et rechutes) signalés à l'OMS en 2014 étaient situés dans la région Afrique. La présente étude avait pour objectif spécifique d'estimer les pertes de produit intérieur brut (PIB) associées aux décès imputables à la TB dans la région Afrique en vue d'utiliser les résultats afin de promouvoir la mise en œuvre de meilleures stratégies de prévention et de lutte contre la tuberculose.

Méthodes: La méthode du coût de la maladie a été utilisée pour estimer les pertes du PIB non lié à la santé associées aux décès imputables à la TB. Les futures pertes du PIB non lié à la santé ont été actualisées au taux de 3 %. L'analyse a été menée pour trois groupes de pays classés par niveau de revenu. Une analyse de sensibilité unidirectionnelle à un taux d'actualisation de 5 % et 10 % a été réalisée afin d'évaluer l'impact sur la perte prévue du PIB non lié à la santé.

Résultats: D'après les estimations, les 753 000 décès imputables à la tuberculose qui se sont produits dans la région Afrique en 2014 entraîneraient une diminution du futur PIB non lié à la santé de 50,4 milliards de dollars internationaux. Une proportion près de 40,8 %, 46,7 % et 12,5 % de cette perte serait issue de pays à revenu élevé et intermédiaire supérieur ou respectivement de pays à revenu intermédiaire inférieur ou faible. La perte totale moyenne du PIB non lié à la santé s'élèverait à 66 872 dollars internationaux par décès imputable à la TB. La perte moyenne du PIB non lié à la santé par décès imputable à la TB s'élevait à 167 592 dollars internationaux pour le groupe 1, 69 808 dollars internationaux pour le groupe 2 et 21 513 dollars internationaux pour le groupe 3.

Conclusion: La tuberculose représente un fardeau économique considérable pour les économies des pays de la région Afrique de l'OMS. Cela implique la nécessité de renforcer la promotion de la mise en œuvre de meilleures stratégies de prévention et de lutte contre la tuberculose et d'aider les pays à mettre un terme à l'épidémie de tuberculose d'ici 2030, comme l'envisage la résolution de l'Assemblée Générale des Nations Unies sur les Objectifs de Développement Durable (ODD).

Translated from English version into French by Eric Ragu, through

TRANSLATORS
WITHOUT BORDERS

Потери производительности, связанные со смертностью от туберкулеза в африканском регионе по данным Всемирной организации здравоохранения

Joses Muthuri Kirigia，Rosenabi Deborah Karimi Muthuri

Аннотация

Краткая информация: В 2014 году почти половина общего количества смертей от туберкулеза произошла в африканском регионе по данным Всемирной организации здравоохранения (ВОЗ). Примерно 21,5 % из 6 060 742 случаев туберкулеза (новых случаев и рецидивов) по данным ВОЗ имели место в 2014 году в Африканском регионе. Конкретная цель данного исследования состояла в том, чтобы оценить будущие потери внутреннего валового продукта (ВВП), связанные со смертностью от туберкулеза в Африканском регионе, для использования в продвижении более эффективных стратегий по профилактике туберкулеза и борьбе с ним.

Методы: Метод стоимости болезни был использован для оценки потерь ВВП, не связанных со здоровьем, из-за смертности от туберкулеза. Будущие потери ВВП, не связанные со здоровьем, дисконтировались по ставке 3 %. Анализ проводился для трех групп населения стран по уровню доходов. Односторонний анализ чувствительности по дисконтным ставкам 5 % и 10 % был проведен для оценки воздействия на ожидаемые потери ВВП, не связанные со здоровьем.

Результаты: Ожидается, что 0,753 миллиона случаев смерти от туберкулеза, которые произошли в Африканском регионе в 2014 году, снизят будущий ВВП, не связанный со здоровьем, на 50,4 млрд международных долларов (Int$). Почти 40,8 %, 46,7 % и 12,5 % этих потерь будут иметь место в странах с высоким уровнем дохода и уровнем дохода выше среднего, или же с низким уровнем дохода и уровнем дохода ниже среднего, соответственно. В среднем общие потери ВВП, не связанные со здоровьем, составят 66 872 Int$ за каждую смерть от туберкулеза. Средние потери ВВП, не связанные со здоровьем, за каждую смерть от туберкулеза, составили 167 592 Int$ для группы 1, 69 808 Int$ для группы 2 и 21 513 Int$ для группы 3.

Заключение: Туберкулез оказывает значительное экономическое бремя на экономику стран африканского региона ВОЗ. Это подразумевает необходимость решительно выступать за применение более эффективных стратегий по профилактике туберкулеза и борьбе с ним и за оказание помощи странам в ликвидации эпидемии туберкулеза к 2030 году, как это предусмотрено в резолюции Генеральной Ассамблеи Организации Объединенных Наций по целям устойчивого развития (ЦУР).

Translated from English version into Russian by Oksana Weiss, through

TRANSLATORS
WITHOUT BORDERS

Pérdida de productividad asociada a muertes por tuberculosis en la Región de África de la Organización Mundial de la Salud

Joses Muthuri Kirigia, Rosenabi Deborah Karimi Muthuri

Resumen

Antecedentes: En el año 2014, casi la mitad de las muertes por tuberculosis en el mundo se produjeron en la Región de África de la Organización Mundial de la Salud (OMS). Aproximadamente 21,5% de los 6.060.742 casos de TB (nuevos y recaídas) que se reportaron a la OMS en el año 2014 fueron en la Región de África. El objetivo específico del presente estudio fue estimar las pérdidas a futuro del producto bruto interno (PBI) que se asocian a muertes por TB en la Región de África para utilizarlas como fundamento en la solicitud de mejores estrategias para prevenir y controlar la tuberculosis.

Métodos: Para estimar las pérdidas de PBI (excluyendo salud) asociadas a muertes por TB se utilizó el método costo-de-enfermedad. Las pérdidas a futuro de PBI no asociado a salud se descontaron en un 3%. El análisis se llevó a cabo para tres grupos de países por ingresos. Se realizó un análisis de sensibilidad unidireccional al 5% y al 10% para evaluar el impacto a esperar de las pérdidas de PBI no asociado a salud.

Resultados: Se espera en el futuro que las 0,753 millones de muertes por tuberculosis que se produjeron en la Región de África en el año 2014 disminuyan el PBI no asociado a salud en 50,4 mil millones de dólares internacionales (Int$). Casi 40,8%, 46,7% y 12,5% de dicha pérdida provendría de países de ingresos altos y medio-altos o de países de ingresos medio-bajos y bajos, respectivamente. La pérdida promedio de PBI no asociado a salud por muertes por tuberculosis sería de Int$ 66.872. La pérdida promedio de PBI no asociado a salud por muerte por TB fue de Int$167.592 para el Grupo 1, Int$69.808 para el Grupo 2 e Int$21.513 para el Grupo 3.

Conclusión: La tuberculosis plantea una carga económica significativa para las economías de los países de la Región de África de la OMS. Esto significa que se necesita abogar enérgicamente por mejores estrategias para prevenir y controlar la tuberculosis y para ayudar a los países a que terminen con la epidemia de tuberculosis para el año 2030, como se visualizó en la resolución sobre los objetivos de desarrollo sostenible en la Asamblea General de las Naciones Unidas.

Translated from English version into Spanish by Maria Alejandra Aguada, through

TRANSLATORS
WITHOUT BORDERS
